# Neuronal Apoptosis and Motor Deficits in Mice with Genetic Inhibition of GSK-3 Are Fas-Dependent

**DOI:** 10.1371/journal.pone.0070952

**Published:** 2013-08-05

**Authors:** Raquel Gómez-Sintes, José J. Lucas

**Affiliations:** 1 Centro de Biología Molecular “Severo Ochoa” (CBM“SO”), CSIC/UAM, Madrid, Spain; 2 Centro de Investigación Biomédica en Red sobre Enfermedades Neurodegenerativas (CIBERNED), Instituto de Salud Carlos III, Madrid, Spain; New York State Institute for Basic Research, United States of America

## Abstract

Glycogen synthase kinase-3 (GSK-3) inhibitors have been postulated as useful therapeutic tools for the treatment of chronic neurodegenerative and neuropsychiatric diseases. Nevertheless the clinical use of these inhibitors has been limited by their common side effects. Lithium, a non-selective GSK-3 inhibitor has been classically administered to treat bipolar patients but its prescription is decreasing due to its frequent side effects such as hand tremor. This toxicity seems to be higher in the elderly and a clinical trial with lithium for Alzheimer’s disease was stopped due to high rate of discontinuation. We have previously described a mechanism for the adverse effects of chronic lithium that involves neuronal apoptosis via Fas signaling. As lithium inhibits many other enzymatic activities such as inositol monophosphatase and histone deacetylase, here we aim to genetically test whether GSK-3 inhibition induces those adverse effects through Fas receptor. For this purpose we took advantage of a transgenic mouse line with decreased GSK-3 activity (Tet/DN-GSK-3 mice) that shows increased rate of neuronal apoptosis as well as motor deficits and brought it to a Fas deficient background (*lpr* mice). We found that apoptosis induced by GSK-3 inhibition was absent in Fas deficient background. Interestingly, motor deficits were also absent in Fas deficient Tet/DN-GSK-3 mice. These results demonstrate that Fas signaling contributes to the neurological toxicity of GSK-3 inhibition and suggest that a combination of GSK-3 inhibitors with blockers of Fas signaling could help to improve the application of GSK-3 inhibitors to clinics.

## Introduction

GKS-3 is involved in many cellular signaling pathways such as the insulin/PI3K or the Wnt pathways and participates in a high number of functions such as metabolism, cell proliferation, cell fate, survival and apoptosis [1], [2], [3], [4], [5]. Besides, it also plays a key role in certain neuronal specific functions like long term potentiation (LTP) and depression (LTD) of synaptic activity [6], [7]. Dysregulation of GSK-3 has been postulated to participate in the etiology of neuropsychiatric or neurodegenerative diseases: bipolar disorder [8], [9], [10], schizophrenia [9], [11], Alzheimer’s disease (AD) [3], [12], [13] or Huntington’s disease [14], [15], [16] and of non-CNS diseases: type 2 diabetes [17] or cancer [18]. Consequently, GSK-3 inhibitors have been postulated as a promising therapeutic tool [19], [20]. Lithium inhibits GSK-3 [21], [22], [23] and this has been postulated to contribute to its therapeutic efficacy [8], [9], [10] but also to its neurological toxicity [24]. Together, this lithium’s adverse effects [25] and those of GSK-3 genetic inhibition [26], [27] warn about possible limitations of GSK-3 inhibitor based therapies. Understanding of the mechanism of this toxicity and of how to counteract it may be a key step for successful therapeutic use of GSK-3 inhibitors.

GSK-3 is implicated in apoptosis but its modulatory effect can be different depending on the specific apoptotic pathway involved: intrinsic (type I) that involves release of cytochrome c and disintegration of mitochondria and extrinsic (type II) apoptosis that occurs upon the activation of death receptors, specifically the TNF receptor family including Fas and TRAIL [28]. Consequently, lithium and other GSK-3 inhibitors are protective towards many apoptotic stimuli that affect mitochondrial integrity but increase apoptosis triggered by TNF [26], [29] or Fas [30]. Conceivably, this may have significant implications on the therapeutic potential of GSK-3 inhibitors.

Mice with conditional expression of a dominant negative form of GSK-3 (Tet/DN-GSK-3 mice) [27] are a useful tool to explore the neurological consequences of chronically decreasing GSK-3 activity in the brain [24]. Tet/DN-GSK-3 mice display increased rate of neuronal apoptosis and impaired motor coordination [27] that might relate to the frequent neurological motor side effects, such as hand tremor, experienced by lithium-treated patients [25]. Interestingly, wild type mice chronically treated with lithium also show increased rate of neuronal apoptosis and a deficit in motor coordination that have been reported to occur through a mechanism involving Fas, as they are absent in Fas-deficient mice (*lpr* mice) [24]. Since lithium inhibits other enzymes like inositol-monophosphatase or histone-deacetylase [31], it cannot be ascertained that this toxicity mechanism is due to GSK-3 inhibition. Here we test if apoptosis and related behavioral consequences due to decreased GSK-3 activity are Fas dependent.

## Materials and Methods

### Animals

Tet/DN-GSK-3 mice in a C57/BL6J background were generated as described previously [27]. Fas-deficient *lpr* mice (C57/BL/6J background) were obtained from Jackson laboratories (B6.MRL-Faslpr/J, stock number: 000482). All mice were housed at the Centro de Biología Molecular “Severo Ochoa” animal facility. Mice were housed four per cage with food and water available *ad libitum* and maintained in a temperature-controlled environment on a 12/12 h light-dark cycle with light onset at 07∶00 h. For behavioral analysis, mice were tested at the age of 2.5–3 months and they were sacrificed upon completion of the battery of tests. For histological studies only male mice were used. For behavioral studies males and females were used indistinctly. Statistical analysis as genotype x treatment interaction was evaluated by two way-ANOVA to rule out any effect of sex. All behavioral studies were performed during light phase.

### Ethics Statement

Animal housing and maintenance protocols followed the guidelines of Council of Europe Convention ETS123, recently revised as indicated in the Directive 86/609/EEC. Animal experiments were performed under protocols (P15/P16/P18/P22) approved by the Centro de Biología Molecular Severo Ochoa Institutional Animal Care and Utilization Committee (Comité de Ética de Exerimentación Animal del CBM, CEEA-CBM), Madrid, Spain.

### PCR

Mice were genotyped by using the following primers:

For detection of the CamKII-tTA (tTA) transgene: primer tTA-C (5′-ACTAAGTCATCGGATGGAGC-3′) and primer tTA-F (5′-CGAAATCGTCTAGCGCGTCGG-3′) that amplify a 592 bp fragment. For detection of the DN-GSK-3 (tetO-R4) transgene: primer GSK-3A (5′- CATGGTCAGGTCATGGATGAGC-3′) and primer GSK-3B (5′-TAATCAGCCACTGATCCACCCAG-3′) that amplify a 642 bp fragment. Amplification protocol used for both combinations was: 5 min at 94°C followed by 30 cycles of 53°C for 1 min, 72°C for 1,5 min, and 94°C for 1 min and finally 72°C for 5 min. For discriminating the *lpr* mutation we used the protocol recommended by Jackson Laboratories with the three following primers: wild type (5′-CAAATCTAGGCATTAACAGTG-3′), mutant (5′-TAGAAAGGTGCACGGGTGTG) and common (5′-GTAAATAATTGTGCTTCGTCAG-3′) that yield a 179 bp band from the wild type allele and a 217 bp band from the mutant allele.

### Western Blot Analysis

Mice were sacrificed using CO_2_ and brains immediately removed and dissected on an ice-cold plate. Whole extracts were prepared by homogenizing the brain areas from right hemisphere in ice-cold extraction buffer consisting of 20 mM HEPES pH 7.4, 100 mM NaCl, 20 mM NaF, 1% Triton X-100, 1 mM sodium orthovanadate, 1 µM okadaic acid, 5 mM sodium pyrophosphate, 30 mM β-glycerophosphate, 5 mM EDTA, and protease inhibitors (2 mM PMSF, 10 µg/ml aprotinin, 10 µg/ml leupeptin and 10 µg/ml pepstatin). Samples were homogenized and centrifuged at 15000 g for 20 min at 4°C. The resulting supernatant was collected, and protein content determined by Bradford assay. Fifteen micrograms of total protein were electrophoresed on 10% SDS-polyacrylamide gel and transferred to a nitrocellulose membrane (Schleicher and Schuell). The experiments were performed using the following primary antibodies: anti-β-gal (Promega, 1∶2000) and anti-β-actin (Sigma, 1∶5000). The membranes were incubated with antibody overnight at 4°C in 5% non-fat dried milk. Secondary antibodies used were polyclonal rabbit anti-mouse immunoglobulins/HRP (DAKO Cytomation) (1∶2000) and ECL detection reagents (Perkin Elmer) were used for immunodetection.

### Immunofluorescence and Immunohistochemistry

Left hemispheres were processed for histology placed in 4% paraformaldehyde in Sorensen's phosphate buffer (PFA) overnight, washed in PBS and then immersed in 30% sucrose in PBS for 72 hr. Once cryoprotected, the samples were included in OCT compound (Sakura Finetek Europe) frozen and stored at −80°C until use. 30 µm sagittal sections were cut on a CM 1950 Ag Protect freezing microtome (Leica) and collected and stored free floating in glycol containing buffer (30% glycerol, 30% ethylene glycol in 0,02 M phosphate buffer) at −20°C.

For immunofluorescence, 30 µm sagittal brain sections were pretreated with 0,1% Triton X-100 for 15 min, 1 M Glycine for 30 min, and blocking solution (1% BSA and 0,1% Triton X-100) for 1 hour. Sections were then incubated overnight at 4°C with primary antibodies in blocking solution at the following concentrations: cleaved-caspase-3 (Cell Signaling Technology, MA) (1∶50), β-gal (Promega) 1∶2000 and NeuN (Chemicon) (1∶100). The following day, sections were washed in PBS. Then sections were incubated with donkey anti-rabbit Alexa 555 (Invitrogen) (1∶500), donkey anti-mouse Alexa 488 (Invitrogen) (1∶1000) and goat anti-mouse Alexa 488 (Invitrogen) (1∶1000) secondary antibodies for 1 hour. Finally, nuclei were counterstained with DAPI (1∶5000, Calbiochem). Sections were mounted on glass slides, coverslipped with Fluorsave (Calbiochem) and maintained at 4°C.

Colocalization of markers was identified by taking successive Alexa 555 and Alexa 488 fluorescent images using a Laser Confocal LSM710 camera (Zeiss) coupled to an inverted microscope AxioObserver (Zeiss).

For immunohistochemistry, brain sections were pretreated for 30 min in 1%H_2_O_2_/PBS followed by 1 h with 1% BSA, 5% FBS and 0.2% Triton X-100 and incubated overnight at 4°C with β-gal (ICN Biomed.-Cappel, 1∶2000) or cleaved-caspase-3 (Cell Signaling, 1∶50) primary antibodies. Finally, brain sections were incubated in avidin-biotin complex using the Elite Vectastain kit (Vector Laboratories). Chromogen reactions were performed with diaminobenzidine (SIGMA*FAST*™ DAB, Sigma) for 10 min. Sections were mounted on glass slides and coverslipped with Fluorosave (Calbiochem). For quantification of cleaved-caspase-3 immunostainings, four male mice per genotype were processed as follows: one every four serial 30 µm-sagittal sections were selected for staining between the 3.00 mm and 0.72 mm planes of the Paxinos and Franklin mouse brain atlas [32]. Round-shape (5–10 µm in diameter) positive cells were quantified with the “Analyze particles” tool of Image J. Non-round shape cells with characteristic apoptotic bodies were detected manually. All analyses were performed blind and results were presented as the estimate of total positive cells in the whole brain structure.

### TUNEL Assay

Sections were processed according to the In Situ Cell Death Detection Kit protocol (POD, Roche). Quantification was performed on 4 male mice per genotype by staining one every four serial 30 µm-sagittal sections spanning from the 3.00 mm to the 0.72 mm planes of the Paxinos and Franklin mouse brain atlas [32]. Round-shape (5–10 µm in diameter) positive cells were quantified with the “Analyze particles” tool of Image J. All analyses were performed blind and results were presented as the estimate of total TUNEL-positive cells in the whole brain structure.

### Behavioral Testing

#### Treadmill gait analysis

Gait analysis was performed using the DigiGait™ system (Mouse Specifics Inc., Boston, MA) [33]. Briefly, digital images of paw placement were recorded at 80 Hz through a clear treadmill from beneath the animal. Mice were tested without pre-training in one session at a treadmill speed of 24 cm/s. Paws were marked with red colorant for better contrast. Plotting the area of each digital paw print imaged sequentially in time provides a dynamic gait signal, representing the temporal record of paw placement relative to the treadmill belt. Swing duration was measured as the time duration of the swing phase, when no paw is in contact with the belt. Paw angle variability is the variability of paw angle, considered as the angle that the paw makes with the long axis of the direction of motion. Stride length variation represents the standard deviation of the stride length for the set of strides recorded (reflecting the dispersion about the average value) [33]. Step angle variation was measured as CV and was calculated using the equation: *100 x standard deviation/mean value* (variability normalized to the mean). Step angle factors both stance width and stride length. Stance asymmetry is the ratio of left hind limb stance to right hind limb. The number of animals used for this test was: wt, n = 14 (5 males/9 females); Tet/DN-GSK-3, n = 12 (4 males/8 females); lpr/+, n = 22 (13 males/9 females); lpr/+;Tet/DN-GSK-3, n = 23 (12 males/11 females); lpr/lpr, n = 15 (11 males/4 females) and lpr/lpr;Tet/DN-GSK-3, n = 9 (5 males/4 females).

#### Vertical pole test

Testing was performed as previously described [34] with minor modifications [27]. The mouse was placed head-upward on the top of a vertical rough-surfaced pole (diameter 1 cm; height 50 cm) and the time taken to descend to the floor was recorded with a maximum duration of 60 s. The number of animals used for this test was: wt, n = 16 (6 males/10 females); Tet/DN-GSK-3, n = 18 (10 males/8 females); lpr/+, n = 28 (16 males/12 females); lpr/+;Tet/DN-GSK-3, n = 23 (12 males/11 females); lpr/lpr, n = 16 (13 males/3 females) and lpr/lpr;Tet/DN-GSK-3, n = 10 (5 males/5 females).

#### Rotarod

Test was performed with accelerating rotarod apparatus (Ugo Basile, Comerio, Italy). Mice were pre-trained during two days at a constant speed, 4 rpm the first day or 8 rpm the second day. Then, rotarod was set to accelerate from 4 to 40 rpm over 5 min and mice were tested four times. During accelerating trials, the latency to fall from the rod was measured. The number of animals used for this test was: wt, n = 16 (6 males/10 females); Tet/DN-GSK-3,n = 19 (10 males/9 females); lpr/+, n = 28 (15 males/13 females); lpr/+;Tet/DN-GSK-3, n = 25 (13 males/12 females); lpr/lpr, n = 16 (12 males/4 females) and lpr/lpr;Tet/DN-GSK-3, n = 10 (5 males/5 females).

#### Open Field

Locomotor activity was measured in clear plexiglas boxes measuring 43.2 cm×43.2 cm, outfitted with photo-beam detectors for monitoring horizontal and vertical activity. Activity levels were recorded with a MED Associates’ Activity Monitor (MED Associates, St. Albans, VT). Locomotor activity data were collected via a PC and was analyzed with the MED Associates’ Activity Monitor Data Analysis software. Mice were placed in a corner of the open-field apparatus and left to move freely. Variables recorded included: resting time (s), ambulatory time (s), vertical/rearing time (s), jump time (s), stereotypic time (s) and average velocity (cm/s). Data were individually recorded for each animal during 15 min.

The number of animals used for this test was: wt, n = 6 (2 males/4 females); Tet/DN-GSK-3,n = 6 (1 male/5 females); lpr/+, n = 11 (7 males/4 females); lpr/+;Tet/DN-GSK-3, n = 14 (8 males/6 females); lpr/lpr, n = 12 (5 males/7 females) and lpr/lpr;Tet/DN-GSK-3, n = 6 (3 males/3 females).

In order to rule out any sex difference statistical analysis was performed evaluating genotype × sex interaction for each parameter by a two way-ANOVA. The following results were obtained: swing (p = 0.001, F = 4.142), stride length variability (p = 0.054, F = 2.221), paw angle variability (p = 0.114, F = 1.805), paw area variability (p = 0.159, F = 1.612), vertical pole (p = 0.823, F = 0.436), rotarod (p = 0.662, F = 0.651), resting time (p = 0.772, F = 0.504), ambulatory time (p = 0.626, F = 0.700), vertical/rearing time (p = 0.999, F = 0.045), jump time (p = 0.878, F = 0.353), stereotypic time (p = 0.904, F = 0.311) and average velocity (p = 0.450, F = 0.935). As no sex differences were found among genotypes, males and females were used indistinctly.

Behavioral tests were performed during three consecutive days as follows: first day, treadmill gait analysis and first training day of rotarod; second day, vertical pole and second training day of rotarod; and third day, four accelerating trials in the rotarod. The first group of animals tested performed an additional open field one day before starting behavioral tests.

#### Statistical analysis

Statistical analysis was performed with SPSS 19.0. Data are presented as mean values ± S.E.M. The normality of the data was analyzed by Shapiro-Wilk test. Statistical analysis of data with a normal distribution was performed following a one way-ANOVA test followed by a DMS or a Bonferroni *post-hoc* test. Statistical significance of non-parametric data was determined by Kruskal-Wallis test when analyzing all experimental groups, followed by a Mann-Whitney U-test for analysis of paired genotypes and Bonferroni correction was applied. Genotype frequencies were analyzed by means of chi-square test. A critical value for significance of p<0.05 was used throughout the study.

## Results

### Tet/DN-GSK-3 Mice in lpr Background are Viable and Grow Normally

**Figure 1:**
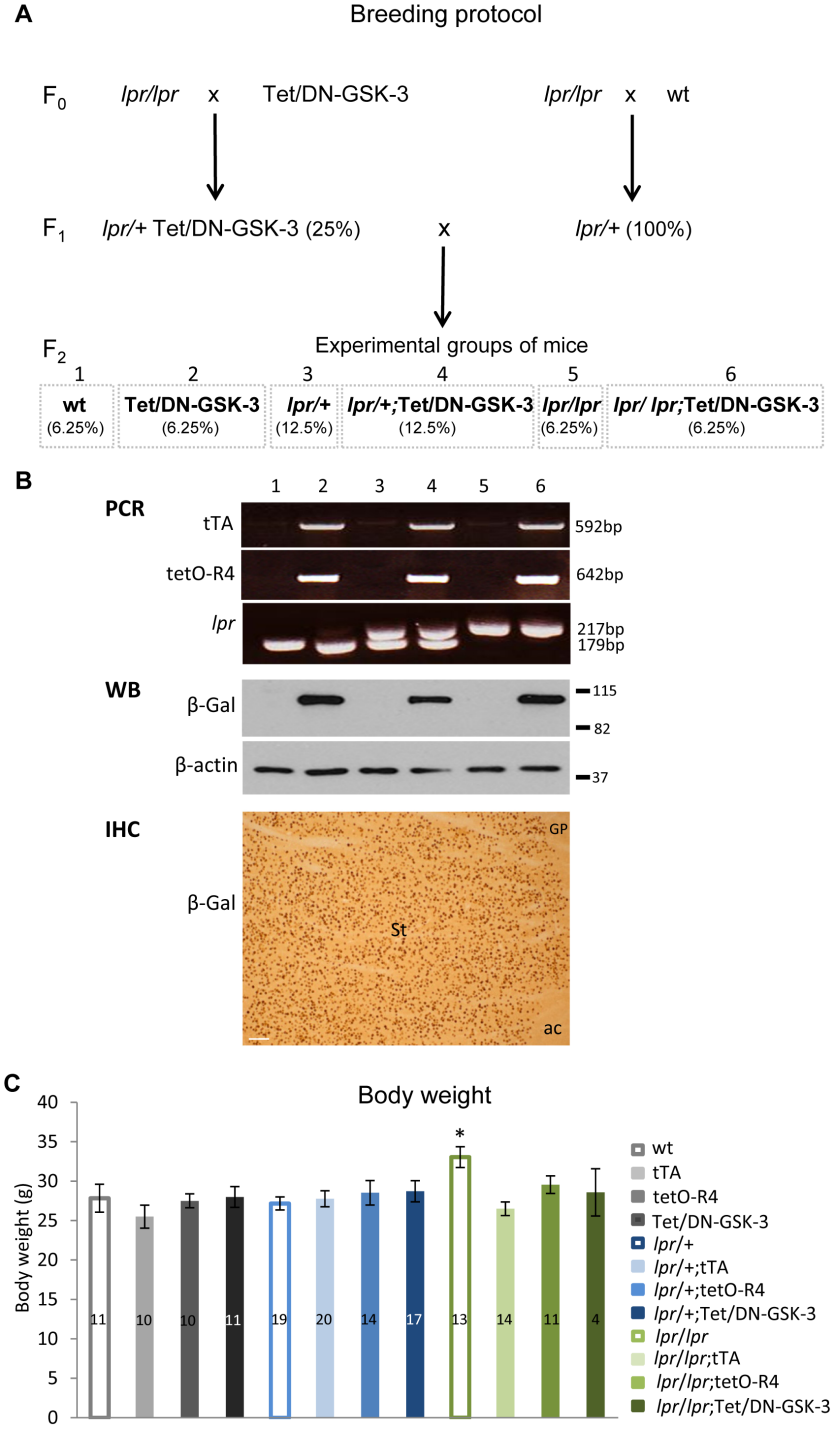
Generation of experimental mice and analysis of body weight. A. Diagram showing the breeding design. First, *lpr*/+;Tet/DN-GSK-3 and *lpr*/+ mice (F1) were obtained by crossing *lpr/lpr* mice with Tet/DN-GSK-3 or wt mice respectively (F0). Then, *lpr*/+;Tet/DN-GSK-3 and *lpr*/+ mice were combined to obtain 12 possible genotypes among which we obtained the 6 experimental groups: wt, Tet/DN-GSK-3, *lpr*/+, *lpr*/+;Tet/DN-GSK-3, *lpr/lpr* and *lpr*/*lpr*;Tet/DN-GSK-3. B. Identification of the different genotypes by PCR to detect the CamKII-tTA and the DN-GSK-3 transgenes and the presence of the *lpr* mutation. Line 1: wt; line 2: Tet/DN-GSK-3; line 3: *lpr*/+; line 4: *lpr*/+; Tet/DN-GSK-3; line 5: *lpr*/*lpr;* line 6: *lpr*/*lpr*; Tet/DN-GSK-3. Analysis of the transgene expression by western blot in striatum the six experimental genotypes and example of immunohistochemistry of the reporter β-gal in striatum (St) of Tet/DN-GSK-3 mice. Scale bar corresponds to 100 µm. ac, anterior commissure; GP, globus pallidus. C. Body weight of all resulting genotypes was measured at the age of 2.5 months. One-way ANOVA test followed by Bonferroni *post-hoc* test was applied to determine the level of significance. The number of analyzed animals is indicated in the graph. Only *lpr/lpr* mice were significantly different than wt mice (* p<0.05).

To explore whether the Fas dependent neuronal death observed in lithium-treated mice [24] could be mimicked by inhibiting GSK-3 via a dominant-negative genetic approach, we decided to generate Tet/DN-GSK-3 mice in Fas-deficient background for subsequent behavioral and brain apoptosis analysis. Mice expressing DN-GSK-3 in forebrain neurons in a tetracycline repressible manner (Tet/DN-GSK-3 mice) were generated as previously described [27] by combining mice expressing tTA under CamKIIα promoter (tTA mice) with mice carrying myc-K85R-GSK-3 and β-Gal sequences fused to a bidirectional tetO promoter (tetO-R4 mice). Fas-deficient mice (*lpr* mice) are naturally deficient in Fas receptor [35]. Double transgenic Tet/DN-GSK-3 mice (that carry both the tTA-CamKIIa transgene, and the TetO-R4 transgene) were then combined with *lpr/lpr* mice as shown in Fig. 1A. Resulting lpr/+;Tet/DN-GSK-3 (F1) were then bred with *lpr*/+ (F1) mice to obtain the six experimental genotypes out of the twelve possible ones (Table 1). For identification of genotypes, three different PCR reactions were performed to detect the CamKII-tTA construct, the GSK-3 construct and the *lpr* mutation (Fig. 1B). To verify expression of the transgene, western blot and immunohistochemistry of the β-gal reporter were also performed (Fig. 1B). Analysis of the frequencies by means of a chi-square test revealed that all genotypes adjusted to the expected Mendelian distribution (Table 1) and they displayed no overt phenotype. They also displayed body weight similar to that of their control wild type (wt) littermates (Fig. 1C) except for *lpr*/*lpr* mice that were bigger than wild type as previously reported [36]. One-way ANOVA test (p = 0.026) followed by Bonferroni *post-hoc* test was applied to determine the level of significance and when males and females were tested separately, a similar effect was found for lpr/lpr mice. However, this difference was not evident in *lpr*/*lpr* mice harbouring any of the Tet/DN-GSK-3 transgenes. For further biochemical, histological and behavioral analysis only wt, Tet/DN-GSK-3, *lpr*/+, *lpr*/+;Tet/DN-GSK-3, *lpr*/*lpr* and *lpr*/*lpr*;Tet/DN-GSK-3 mice were used and they were analyzed at the age of 2.5–3 months.

**Table 1:** Genotype frequencies.

Nomenclature	**wt**	tTA	tetO-R4	**Tet/DN-GSK-3**	***lpr*** **/+**	*lpr*/+;tTA	*lpr*/+;tetO-R4	***lpr*** **/+;Tet/DN-GSK-3**	***lpr*** **/** ***lpr***	*lpr*/*lpr*;tTA	*lpr*/*lpr*;tetO-R4	***lpr*** **/** ***lpr*** **;Tet/DN-GSK-3**
lpr locus	+/+	+/+	+/+	+/+	lpr/+	lpr/+	lpr/+	lpr/+	lpr/lpr	lpr/lpr	lpr/lpr	lpr/lpr
Tet/DN-GSK-3 transgenes	–	tTA	tetO-R4	tTA+tetO-R4	–	tTA	tetO-R4	tTA+tetO-R4	–	tTA	tetO-R4	tTA+tetO-R4
Distribution/frequencies												
N	28	32	20	21	67	37	34	37	27	21	13	10
Expected frequencies	6.25	6.25	6.25	6.25	12.5	12.5	12.5	12.5	6.25	6.25	6.25	6.25
Observed frequencies	8.07	9.22	5.76	6.05	19.31	10.66	9.8	10.66	7.78	6.05	3.75	2.88

### Absence of Apoptosis in Cortex and Striatum of Fas Deficient-Tet/DN-GSK-3

**Figure 2:**
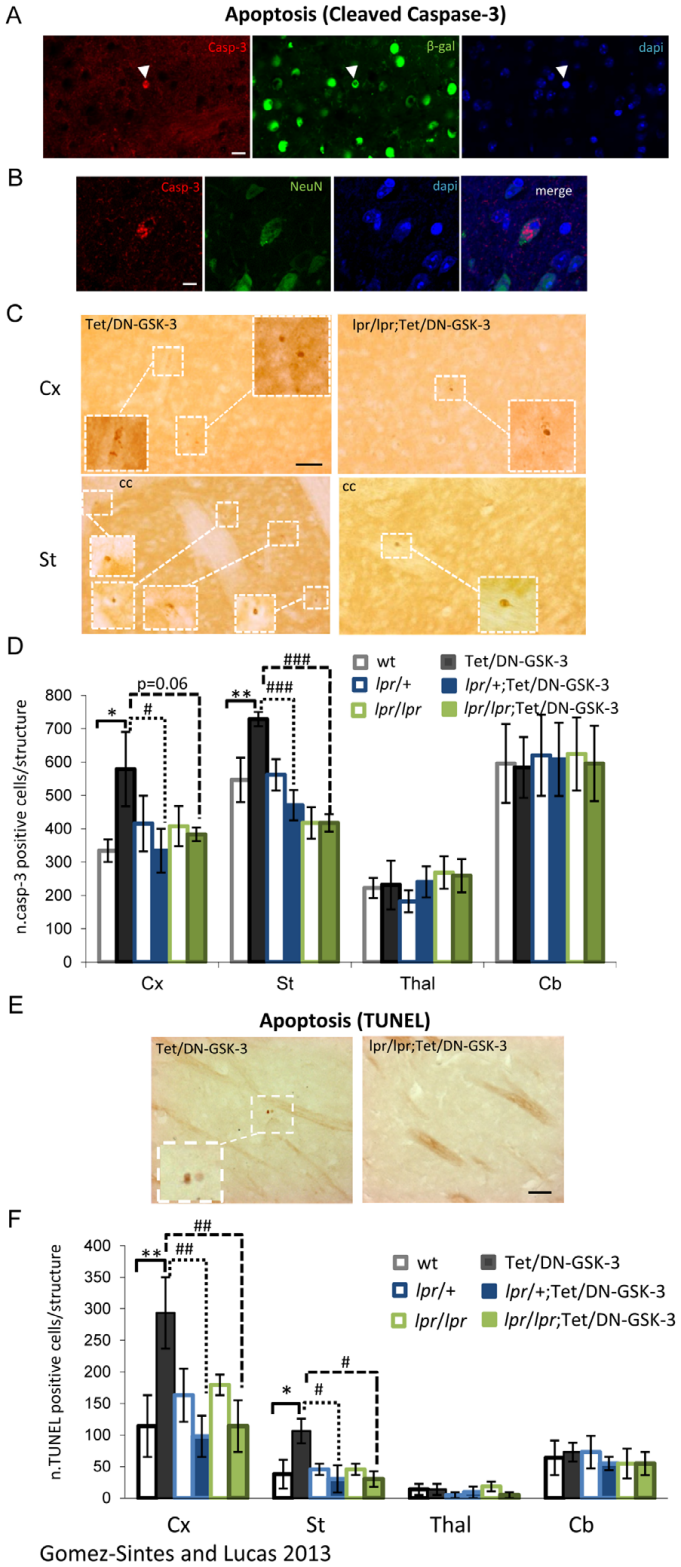
Analysis of apoptotic levels in experimental mice. A. Double labeling immunofluoresce with anti-cleaved caspase-3 (red) and β-gal (green) antibodies. Scale bar in left panel corresponds to 20 µm. B. Double labeling immunofluoresce with anti-cleaved caspase-3 (red) and NeuN (green) antibodies. Right panel shows a merged image of caspase-3, NeuN and dapi counterstaining. Scale bar in left panel corresponds to 10 µm. C. Immunohistochemistry with anti-cleaved caspase-3 antibody. Left panels correspond to Cx (upper) and St (lower) of Tet/DN-GSK-3. Right panels correspond to Cx (upper) and St (lower) of *lpr*/*lpr*;Tet/DN-GSK-3. Scale bar in upper left panel corresponds to 50 µm. D. Quantification of caspase-3 positive cells in cortex (Cx), striatum (St), thalamus (Thal) and cerebellum (Cb) of wt, Tet/DN-GSK-3, *lpr*/+, *lpr*/+;Tet/DN-GSK-3, *lpr/lpr* and *lpr*/*lpr*;Tet/DN-GSK-3 mice (4 male mice per genotype). For quantification, one every four serial 30 µm-sagittal sections were selected for staining between the 3.00 mm and 0.72 mm planes of the Paxinos and Franklin mouse brain atlas [32]. Then, the total number of positive cells was extrapolated for the whole structure. E. TUNEL staining. Low magnification pictures were captured at 20×, insets were taken at 40×. Representative images correspond to St of Tet/DN-GSK-3 (left panel) and *lpr*/*lpr*;Tet/DN-GSK-3 (right panel). Scale bar corresponds to 50 µm. F. Quantification of TUNEL positive cells in cortex (Cx), striatum (St), thalamus (Thal) and cerebellum (Cb) of wt, Tet/DN-GSK-3, *lpr*/+, *lpr*/+;Tet/DN-GSK-3, *lpr/lpr* and *lpr*/*lpr*;Tet/DN-GSK-3 mice. Quantification was performed as for Caspase-3 quantification. The number of animals included in this analysis is 4 male animals per genotype. Data are presented as the mean ± s.e.m. number of positive cells per 30 µm section for each structure. Statistical analysis of cleaved caspase-3 immunohistochemistry and TUNEL was performed following a one way-ANOVA test followed by a LSD *post-hoc* test. *,# p<0.05; **,## p<0.01; ***,### p<0.001. * for wt vs. Tet/DN-GSK-3; # for Tet/DN-GSK-3 vs. *lpr*/+;Tet/DN-GSK-3 and *lpr*/*lpr*;Tet/DN-GSK-3 mice**.**

For the analysis of apoptosis, immunofluorescence and immunohistochemistry against cleaved (activated) caspase-3 and TUNEL staining were performed in the six experimental groups of mice at the age of 2.5–3 months (Fig. 2). TUNEL is a common method for detecting DNA fragmentation that results from apoptotic signaling cascades and caspase 3 is one the major caspases activated during the execution phase of apoptosis. First, to verify that the cells undergoing apoptosis express the transgene, we performed double immunofluoresce for caspase-3 and the reporter β-gal (Fig. 2A). As described in our previous report [27], double immunofluorescence with caspase-3 and NeuN (neuronal marker) antibodies revealed an increase in the rate of apoptosis in Tet/DN-GSK-3 mice respect to wild type mice with the majority of apoptotic cells being neurons (Fig. 2B). For a more precise quantification and comparison among the experimental groups with varying *lpr* genotypes, immunohistochemistry of cleaved caspase-3 was performed (Fig. 2C). Counting of positive caspase-3 cells confirmed the previously reported increased rate of apoptosis in cortex (Cx) and striatum (St) of Tet/DN-GSK-3 mice respect to their corresponding control wt littermates (wt vs. Tet/DN-GSK-3: 334.24±33.61 vs. 578.81±111.68 positive cells in Cx, p = 0.023; 546.52±66.75 vs. 728.70±21.47 positive cells in St, p = 0.01) (Fig. 2C–D). As expected, no effect in the number of caspase-3 positive cells was detected in brain regions that do not express the DN-GSK-3 transgene (TetO-R4 transgene) such as the thalamus or the cerebellum. Interestingly, levels of cortical and striatal apoptosis in Fas-deficient-Tet/DN-GSK-3 mice, either *lpr*/+;Tet/DN-GSK-3 or *lpr*/*lpr*;Tet/DN-GSK-3, were similar to control littermates (Tet/DN-GSK-3 vs. *lpr*/+;Tet/DN-GSK-3: p = 0.023 in Cx, p = 0.001 in St; Tet/DN-GSK-3 vs. *lpr/lpr*;Tet/DN-GSK-3: p = 0.062 in Cx, p<0.000 in St). Therefore indicating that apoptosis resulting after sustained inhibition of GSK-3 in striatum and cortex is Fas dependent. Equivalent results were obtained when apoptosis was analyzed by TUNEL method (wt vs. Tet/DN-GSK-3: p = 0.007 in Cx, p = 0.01 in St; Tet/DN-GSK-3 vs. *lpr*/+;Tet/DN-GSK-3: p = 0.004 in Cx, p = 0.005 in St; Tet/DN-GSK-3 vs. *lpr/lpr*;Tet/DN-GSK-3: p = 0.007 in Cx, p = 0.005 in St). (Fig. 2E–F, statistical analysis of cleaved caspase-3 immunohistochemistry and TUNEL was performed following a one way-ANOVA test followed by a LSD *post-hoc* test). Furthermore, since the absence of DN-GSK-3 mediated apoptosis is not only seen in homozygous *lpr*/*lpr* background but also in the heterozygous *lpr*/+ background, just a partial attenuation of Fas-dependent signaling is sufficient to preclude the effect of DN-GSK-3 on apoptosis induction.

### Absence of Motor Deficits in Tet/DN-GSK-3 Mice in *lpr* Background

**Figure 3:**
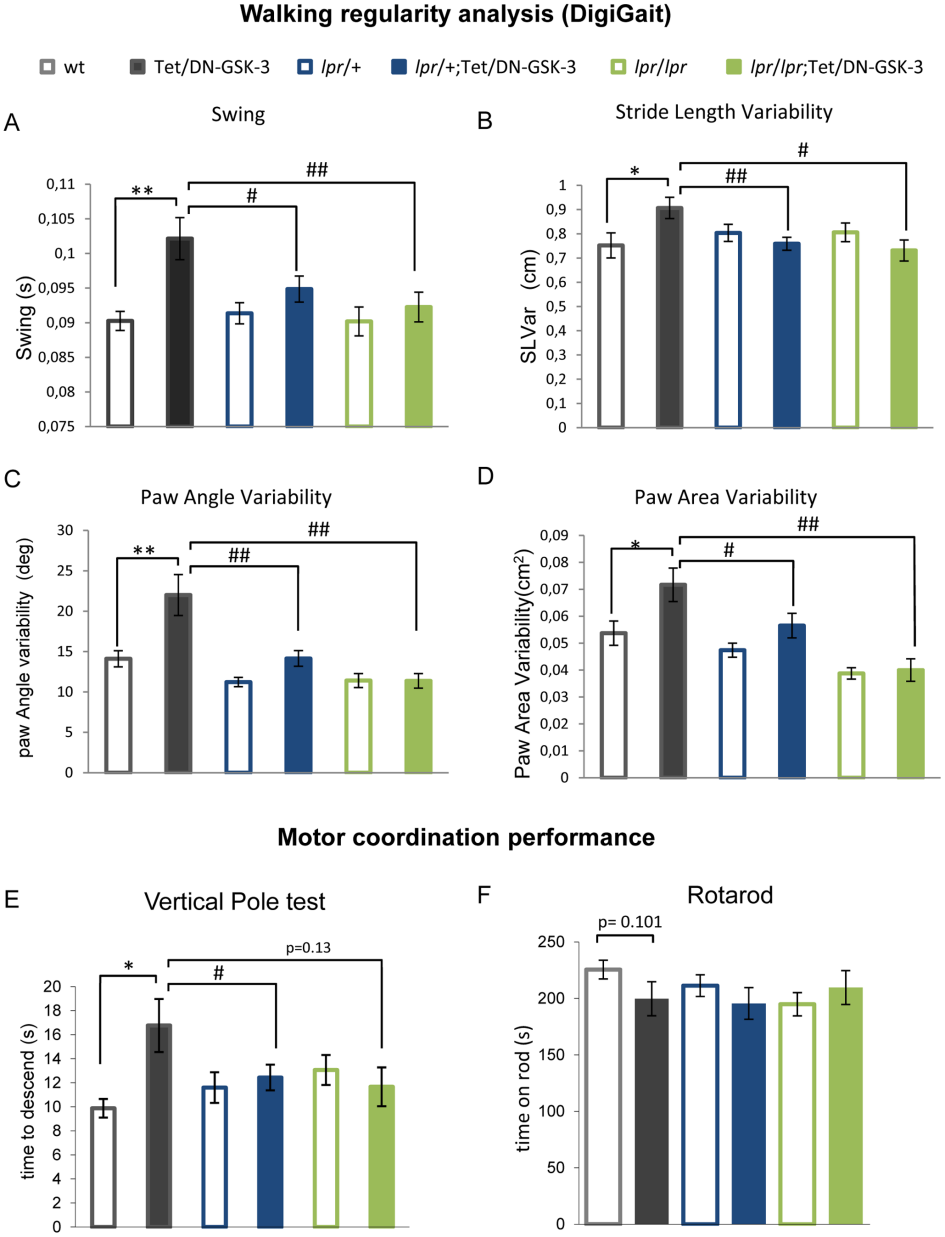
Motor deficits in Tet/DN-GSK-3 mice are Fas dependent. A–D. *Analysis of gait parameters by using the DigiGait apparatus.* Analysis of swing duration (A), stride length variability (B), paw angle variability (C) and paw area variability (D) of experimental mice (wt, n = 13; Tet/DN-GSK-3, n = 12; *lpr*/+, n = 21; *lpr*/+;Tet/DN-GSK-3, n = 21; *lpr/lpr*, n = 14 and *lpr*/*lpr*;Tet/DN-GSK-3, n = 8). Statistical analysis of swing duration, stride length variability and paw angle variability was performed by applying one way-ANOVA test followed by a LSD *post-hoc* test. Statistical analysis of paw area variability was performed using Kruskal-Wallis test for no parametric distribution of data. Then paired genotypes analysis was performed following a Mann-Whitney U-test and Bonferroni correction was applied. *,# p<0.05; **,## p<0.01; ***,### p<0.001; * for wt vs. Tet/DN-GSK-3; # for Tet/DN-GSK-3 vs. *lpr*/+;Tet/DN-GSK-3 and *lpr*/*lpr*;Tet/DN-GSK-3 mice**.** E-F. *Analysis of motor coordination.* E. Time to descend in vertical pole test. (wt, n = 16; Tet/DN-GSK-3, n = 18; *lpr*/+, n = 28; *lpr*/+;Tet/DN-GSK-3, n = 23; *lpr/lpr*, n = 16 and *lpr*/*lpr*;Tet/DN-GSK-3, n = 10). Data are presented as the mean ± s.e.m. time to descend the pole. Statistical analysis was performed applying a one way-ANOVA test followed by a LSD *post-hoc* test. *,# p<0.05. F. Performance in the third and fourth accelerating trials represented as mean ± s.e.m. time on rod (wt, n = 16; Tet/DN-GSK-3,n = 19; *lpr*/+, n = 28; *lpr*/+;Tet/DN-GSK-3, n = 25; *lpr/lpr*, n = 16 and *lpr*/*lpr*;Tet/DN-GSK-3, n = 10). Statistical analysis was performed using Kruskal-Wallis analysis for no parametric distribution of data. Then paired genotypes analysis was performed following a Mann-Whitney U-test.

Increased apoptosis in Tet/DN-GSK-3 is more prominent in striatum and cortex which are part of the basal ganglia circuit, involved in motor control. Accordingly, Tet/DN-GSK-3 mice have been reported to show deficits in motor tasks like the vertical pole and the footprint tests [27]. Since we have found no evidence of increased neuronal death in striatum and cortex of Tet/DN-GSK-3 mice with Fas deficiency (*lpr*/+ or *lpr*/*lpr*;Tet/DN-GSK-3), we wondered whether motor deficits observed in Tet/DN-GSK-3 mice would also be absent in Fas-deficient Tet/DN-GSK-3 mice. To that end, the six experimental groups of mice were subjected to several behavioral tests that detect potential deficiencies in motor tasks. First we performed DigiGait test, which measures footprint pattern and other parameters of walking regularity (Fig. 3A–D). In good agreement with our previous report [27], Tet/DN-GSK-3 mice subjected to analysis of DigiGait parameters showed multiple gait abnormalities such as increased swing duration (ANOVA, p = 0.006, F = 3.374; Fig. 3A), increased variability in stride length (ANOVA, p = 0.043, F = 2.342; Fig. 3B), increased paw angle variability (ANOVA, p = 0.000, F = 10.306; Fig. 3C) and increased paw area variability (Kruskal-Wallis, p = 0.000; Fig. 3D) as compared to wt mice. Interestingly, *lpr*/+;Tet/DN-GSK-3 and *lpr/lpr*;Tet/DN-GSK-3 showed no abnormalities compared with their respective controls. Statistical analysis of swing duration, stride length variability and paw angle variability was performed by applying one way-ANOVA test followed by a LSD *post-hoc* test while analysis of paw area variability was performed using Kruskal-Wallis test for no parametric distribution of data. Then paired genotypes analysis was performed following a Mann-Whitney U-test and Bonferroni correction was applied. In summary, these results indicate that the increased variability in walking regularity found in Tet/DN-GSK-3 mice is reduced in Fas-deficient backgrounds.

We then performed tests of striatal-dependent motor coordination like the vertical pole and the rotarod tests (Fig. 3E–F). In good agreement with the above shown results, we found significantly increased time to descend the vertical pole for Tet/DN-GSK-3 mice with respect to their control wt littermates (wt vs. Tet/DN-GSK-3: 17±2.2s vs. 10±0.8s, ANOVA: p = 0.036, F = 2.485). Interestingly, the difference was smaller and not significant between *lpr*/+;Tet/DN-GSK-3 (12±1.1s) or *lpr*/*lpr*;Tet/DN-GSK-3 (12±1.6s) and their respective controls (Fig. 3E). Statistical analysis was performed applying a one way-ANOVA test followed by a LSD *post-hoc* test. The accelerating rotarod is another test that detects striatal dependent deficits in motor coordination. This test measures the time spent by the mouse on a rotating cylinder (rod) as the speed of rotation is accelerating from 4 to 40 r.p.m. Results of rotarod test were represented as average of third and fourth trials (Fig. 3F). Despite a tendency to decreased time on rod of Tet/DN-GSK3 mice (wt vs. Tet/DN-GSK-3: 225±8.26 vs. 199±15.1, p = 0.101 in Kruskal-Wallis analysis followed by Mann-Whitney U-test.), no significant difference was observed for any group. Finally, to verify that these animals do not show impairment in general locomotive behavior, they were subjected to open field test (Fig. S1) and several parameters as inactivity time (p = 0.289, F = 1.277; Fig. S1A), horizontal activity (p = 0.116, F = 1.876; Fig. S1B), vertical activity (p = 0.0.064, F = 2.511; Fig. S1C), jump time (p = 0.788; Fig. S1D), stereotypic movements (p = 0.0.271, F = 1.32; Fig. S1E) or average velocity (p = 0.098, F = 1.982; Fig. S1F), were measured and no differences among genotypes were found. Statistical analysis was performed applying a one way-ANOVA test except for jump time for that a Kruskal-Wallis test was applied.

## Discussion

By combining conditional transgenic mice with neuronal expression of a dominant negative form of GSK-3 (Tet/DN-GSK-3 mice) and *lpr* mice, we have generated mice with sustained inhibition of GSK-3 in a Fas-deficient background for analysis of apoptosis in brain and for behavioral characterization of their ability to carry out motor tasks. Unlike Tet/DN-GSK-3 mice that show increased apoptosis in cortex and striatum [27], *lpr*/+;Tet/DN-GSK-3 and *lpr/lpr*; Tet/DN-GSK-3 mice showed levels of neuronal apoptosis similar to those found in wild type mice. In addition, analysis of motor coordination in tests of walking regularity, in the vertical pole test and in the rotarod test revealed that the motor deficits previously reported in Tet/DN-GSK-3 mice [27] were no longer evident when genetic inhibition of GSK-3 occurs in a Fas-deficient background.

Lithium prescription to bipolar disorder patients has decreased in the last years due to its common side effects [37], [38]. Understanding the molecular mechanism by which lithium exerts its neurological toxicity may lead to strategies to overcome its side-effects. In this regard, inhibition of GSK-3 by lithium has been postulated to contribute not only to its therapeutic efficacy [8], [9], [10] but also to its neurological toxicity [24]. Since increased GSK-3 activity is believed to contribute to the etiology of other pathologies such as AD, lithium has been postulated as a possible therapy for AD [19]. However, despite potential benefits of lithium for amnestic minimal cognitive impairment in a recent trial [39], clinical trials for AD have been hampered by high rates of discontinuation due to lithium’s adverse effects that is even more prominent in the elderly [40]. Therefore, toxicity of lithium therapy is a problem that extends beyond mood disorders. Regarding the molecular basis of lithium toxicity, we have recently reported a mechanism for the neurological toxicity of chronic lithium in mice that is dependent on Fas signaling [24]. As mentioned, lithium also inhibits other enzymatic activities such as inositol monophosphatase and histone deacetylase [31]. Although this study does not provide direct evidence that decreasing Fas would decrease toxicity associated with lithium treatment, the results reported here demonstrate that neuronal apoptosis and motor deficits caused by sustained GSK-3 inhibition are Fas dependent. It is therefore conceivable that Fas signaling modulating drugs could be used in the future to improve clinical use of lithium not only for bipolar disorder but also for AD. Besides, more selective GSK-3 inhibitors are currently under development [19], [41], and, similar to lithium, they are known to attenuate neuronal loss in AD animal models with increased GSK3 activity (such β-Amyloid infusion) but also to induce neuronal apoptosis when administered alone to wild type animals without any GSK-3 increasing stimulus [42]. Therefore, modulation of Fas signaling might contribute to the successful application of selective GSK-3 inhibitors to clinics in general.

It is worth noting that factors that attenuate Fas signaling have been suggested *per se* as a potential avenue for therapeutic intervention for AD [43] since increased levels of Fas protein have been reported in the brain and cerebrospinal fluid of AD patients [44], [45] and the *Fas* gene is located in the 10q24.1 region showing linkage to late onset AD [46], [47] with polymorphisms in *Fas* having shown association with AD progression [48], [49]. What would increase the chances of success of a combination of GSK-3 inhibitors and Fas signaling-blockers for AD.

Apart from the mentioned Fas-dependent mechanism for lithium- or genetic GSK-3 inhibition-induced apoptosis there is another reported mechanism by which decreased GSK-3 activity could contribute to exacerbate apoptosis through the extrinsic pathway [50]. More precisely, GSK-3 has been reported to act at an intracellular juxtamembrane location in association with DDX3, the cellular inhibitor of apoptosis protein-1 (cIAP-1) and any of the death receptors to form an antiapoptotic complex. When GSK-3 activity is blocked, stimulation of death receptor potentiates caspase-3 activation [50]. Therefore and in regard to the other above mentioned mechanisms requiring Fas activation, both mechanisms converge in the activation of a death receptor. As in our present study we have blocked apoptosis at the level of Fas (by using Fas-deficient mice), we cannot conclude if stimulation of the receptor or disassembly of the antiapoptotic complex, is predominant. Further experiments specifically aiming blockade of Fas signaling upstream or at the inhibitory complex would be needed to elucidate the contribution of both proposed mechanisms to apoptosis.

One interesting feature of the results reported here is that heterozygous *lpr* modification of Fas is sufficient to correct both the apoptotic and the motor phenotypes of Tet/DN-GSK-3 mice. Therefore, provided the case that pharmacological inhibitors of Fas are developed in the future to facilitate lithium- of more selective GSK-3 inhibitor-based therapies, it is possible that just a partial attenuation of Fas-dependent signaling might sufficient to preclude the effect of GSK-3 inhibitors on apoptosis induction. However, we should also keep in mind the potential drawbacks of prospective Fas inhibiting therapies as Fas signaling is required for many aspects of brain physiology such as neural progenitor survival [51].

An open question remains regarding the results reported here as whether the apoptosis observed in Tet/DN-GSK-3 mice and that disappears in the absence of fully functional Fas receptors is required for the motor phenotype that also gets normalized in a Fas deficient background. In fact, despite higher than in wild type mice, apoptotic rate in Tet/DN-GSK-3 mice is still low and does not produce a visible atrophy of affected structures. Alternatively, the effect in motor coordination could be caused not by the loss of a limited number of neurons in the circuit but by an alteration in the physiology of the global brain structure or circuit. In fact, our group has previously demonstrated that in a mouse model of striatal degeneration (HD94 mice) alterations in motor coordination occur in the absence of striatal cell loss, presumably by dysfunction of striatal neurons [52]. It is therefore possible that Fas signaling may also affect physiology of healthy neuronal circuits [53], [54], [55]. Thus an excessive Fas-FasL signaling could result in apoptosis of a limited number of neurons and in a more widespread dysregulation of neuronal physiology thus contributing to the behavioral consequences induced by GSK-3 inhibition.

In summary, here have genetically proven that neuronal apoptosis and motor deficits caused by sustained GSK-3 inhibition are Fas dependent. This might have important implications to enable combined therapies that could facilitate the clinical use of lithium and or more selective GSK-3 inhibitors not only for bipolar disorder but also for other diseases in which excess GSK-3 activity is believed to contribute to pathogenesis.

## Supporting Information

Figure S1 **All genotypes show similar general locomotive behavior.** Analysis of several parameters in open field test. Analysis of inactivity time (A), horizontal activity (B), vertical activity (C), jump time (D), stereotypic movements (E) or average velocity (F) was performed and no differences among genotypes were found. Statistical analysis was performed applying a one way-ANOVA test except for jump time for that a Kruskal-Wallis test was applied.
(TIF)
