# Supplementary figures and images for: Neuronal Apoptosis and Motor Deficits in Mice with Genetic Inhibition of GSK-3 Are Fas-Dependent
Source: PLoS One. 2013 Aug 5;8(8):e70952. doi: 10.1371/journal.pone.0070952 (PMC3734180; doi:10.1371/journal.pone.0070952)

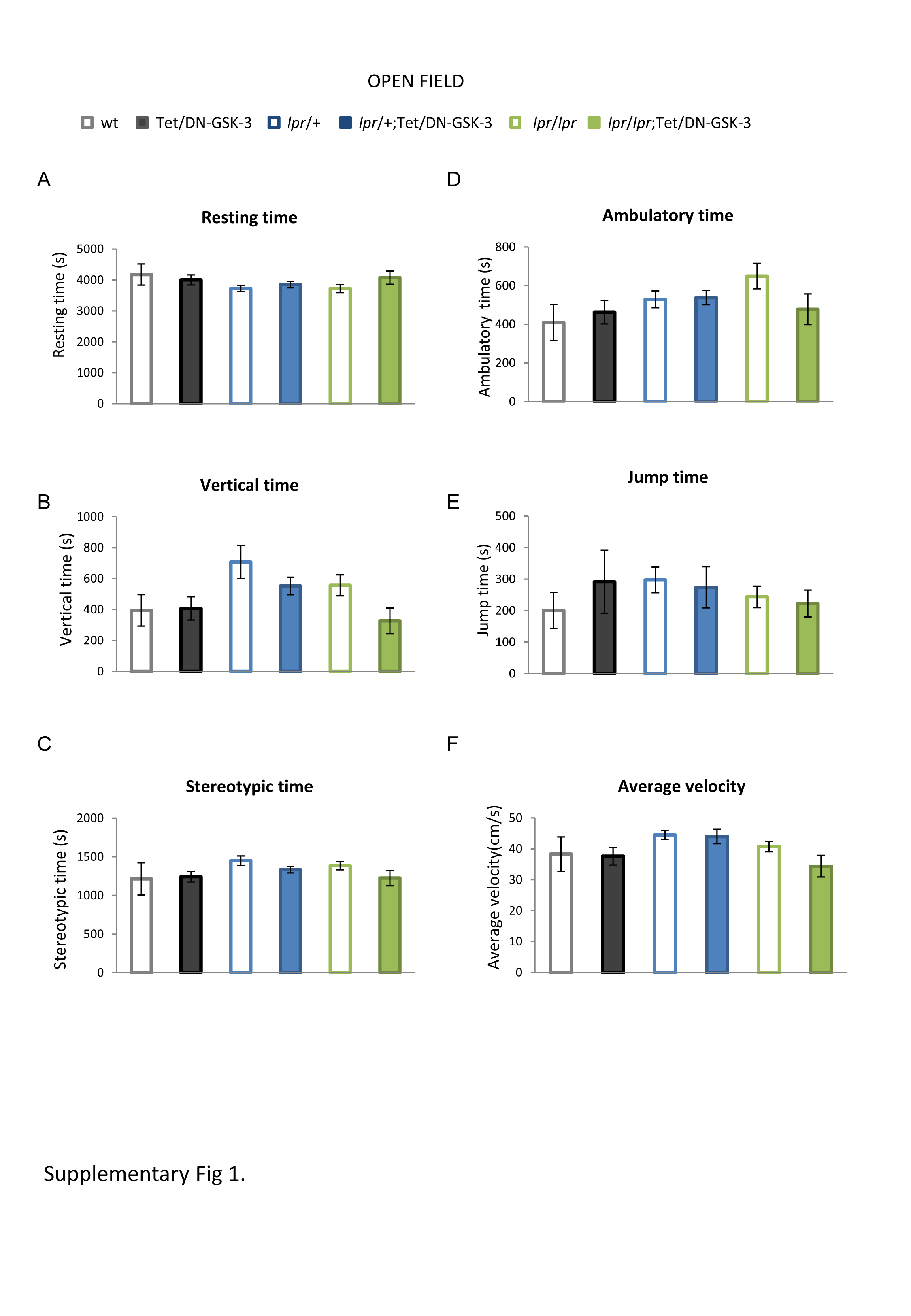

Supplement: Figure S1 — All genotypes show similar general locomotive behavior. Analysis of several parameters in open field test. Analysis of inactivity time (A), horizontal activity (B), vertical activity (C), jump time (D), stereotypic movements (E) or average velocity (F) was performed and no differences among genotypes were found. Statistical analysis was performed applying a one way-ANOVA test except for jump time for that a Kruskal-Wallis test was applied. (TIF) [file pone.0070952.s001.tif]
